# DC/TMD Examiner Protocol: Longitudinal Evaluation on Interexaminer Reliability

**DOI:** 10.1155/2018/7474608

**Published:** 2018-09-26

**Authors:** Marit Slåttelid Skeie, Paula Frid, Manal Mustafa, Jörg Aßmus, Annika Rosén

**Affiliations:** ^1^Department of Clinical Dentistry, Pediatric Dentistry, The Faculty of Medicine, University of Bergen, Bergen, Norway; ^2^Department of Otorhinolaryngology, Division of Oral and Maxillofacial Surgery, University Hospital North Norway and Public Dental Service Competence Centre of North Norway and Department of Clinical Medicine, Faculty of Health Sciences, The Arctic University of Norway, Tromsø, Norway; ^3^Oral Health Centre of Expertise in Western Norway, Hordaland, Norway; ^4^Centre for Clinical Research, Haukeland University Hospital, 5021 Bergen, Norway; ^5^Department of Clinical Dentistry, Division of Oral and Maxillofacial Surgery, The Faculty of Medicine, University of Bergen, Bergen, Norway

## Abstract

**Objectives:**

The objectives of this study were to assess the interexaminer agreement between one “reference” (gold standard) and each of two examiners, using the DC/TMD examination method, Axis I and to evaluate whether a recalibration changed reliability values.

**Methods:**

Participants (4 healthy and 12 TMD patients) in 2013 underwent a clinical examination according to DC/TMDs, Axis I. In 2014, additionally 16 participants (4 healthy and 12 TMD patients) were recruited. Two trainee examiners (one more experienced) and one “reference examiner” (gold standard) at both sessions assessed the participants. Calibration preparation (2013): The clinical protocol was sent to the trainee examiners with a request that its verbal commands should be learned by heart. An eight-hour-course was provided on the day preceding the examination session day. Recalibration preparation (2014): The same examiners in advance to this year's examination session were also asked to recapture the protocol's instructions (verbal commands to be learned by heart) and go through the information from the 2013 course and encouraged to contact by e-mail in case of unclear subjects. At a meeting prior to the examination session, they were also given the opportunities to ask questions. The interexaminer agreements in 2013 and 2014 between the “reference” and each examiner were analysed using Bland–Altman plots, intraclass correlation coefficient, Cohen's kappa, and consistency values.

**Results:**

For the majority of the gathered data, no clear change of agreement between 2013 and 2014 could be observed, and only one muscle zone in 2014 could show any clear difference in agreement between the examiners.

**Conclusions:**

No clear and consistent difference in the level of agreement between the two examiners could be observed, although one was more experienced than the other. Likewise, for most components of the DC/TMD tool, recalibration of examiners did not change the reliability findings.

## 1. Introduction

The temporomandibular disorders (TMDs) and orofacial pain affect around 10–15% of adults [1]. The annual incidence of first-onset TMDs, based on a prospective study, has been reported to be almost 4% [2], meaning that of 100 TMD-free people enrolled, nearly four persons per year will develop the disorder. In the Scandinavian countries, studies have documented pain-related TMDs among adolescents to be 4–7% [3–5], and according to the DC/TMD criteria and examination protocol, to be as high as 12% [4]. Although the disorder may impact the patient's quality of life negatively [6], not all patients receive sufficient and appropriate treatment through the dental health care system [7]. Whether the low provision of treatment is due to under- or misdiagnoses has to be further investigated. What is certain is that the many different diagnostic systems for identifying TMDs contribute to difficulties in agreeing on consistent diagnosis.

The most internationally used diagnostic tools during the last two decades have been the Research Diagnostic Criteria for TMD (RDC/TMD) [8] and the TMD classification according to the American Academy of Orofacial Pain [9], but in 2014, a new diagnostic classification system, the Diagnostic Criteria for Temporomandibular Disorders (DC/TMD), was launched, developed from RDC/TMD [10]. Some of the reasons for updating the RDC/TMDs were that its application was found impractical for use in clinical settings, there was a need to update definitions of TMD subtypes [11, 12], and there was a need for instructions with clear stipulation of specifications in the examination procedures [13]. The goal behind this was to agree on a diagnostic tool for wide use in clinical and research settings [14]. The DC/TMD system has also increasingly gained ground.

The DC/TMDs includes two components, Axis I and Axis II. The Axis I protocol is used for screening and differentiation of the most common pain-related TMDs and also for intra-articular disorders. For TMJ intra-articular disorders, Axis I is appropriate for screening purposes, but not for a definitive diagnosis. To reach a diagnosis, different types of imaging are often needed, such as magnetic resonance imaging (MRI) or computed tomography (CT/CBCT). The Axis II protocol is used to assess jaw physical functioning and to screen behavioural and additional psychosocial status [14].

An important prerequisite, emphasized by the World Health Organization in all oral health survey final reports, is to focus on reliability in the examination process [15]. Without training and calibration, experienced clinicians show low measurement reliability [16]. There are several studies evaluating the reliability and validity of different TMD diagnostic tools [16–23]. In this literature, training and also recalibration have been considered important for improving interexaminer reliability [17]. As far as we know, only a few studies have focused on the reliability of the clinical use of Axis I of the DC/TMDs. Schiffman and Ohrbach [14] have reported Axis I diagnostic criteria for temporomandibular pain-related disorders to have acceptable validity, but the most common pain-related TMJ intra-articular disorders, to be appropriate for screening purposes only. Furthermore, Leskinen et al., who reported on a Finnish version of Axis I DC/TMD clinical diagnoses, have demonstrated sufficiently high reliability for pain-related TMD diagnoses [24]. Graue and colleagues, who estimated the prevalence among Norwegian adolescents using DC/TMDs, also found acceptable clinical interexaminer results [4].

Hitherto, we have found no study using DC/TMDs that focuses on whether recalibration has an effect on reliability. An effect of a prior DC/TMD training course for examiners on reliability, however, has been investigated by Brazilian researchers. They found that the diagnostic reliability of formal DC/TMD training and calibration vs. DC/TMD self-instruction, gave similar values, except for subgroups of myalgia [25].

Arriving at reliable diagnoses is critical and for a relatively new diagnostic tool like DC/TMDs, more research should be given priority. The objective of this study was therefore to assess the interexaminer agreement between one “reference examiner” (gold standard) and each of two trainee examiners, using the DC/TMD examination method, Axis I and to evaluate whether a recalibration changed reliability values.

## 2. Materials and Methods

The null hypothesis to be tested was that there was no difference in reliability values at Time point 1 and Time point 2.

The study protocol was sent to the Regional Committee for Medical Research Ethics in Aarhus, Denmark, for approval. According to the committee's evaluation, the work was accepted as a type of reliability study since identification data, such as participants' names and unique personal identification numbers, were not obtained. In advance of the study, all study participants signed an informed consent.

The study was performed at the Section of Clinical Oral Physiology, Department of Dentistry, Aarhus University, Denmark. The reason why the researchers chose Denmark and not Norway when they conducted the study was due to the fact that in 2013, no course in DC/TMDs was available in Norway. Two independent exercises in DC/TMDs were conducted in 2013 (Sept. 3-4, 2013: Time point 1) and 2014 (June 19, 2014: Time point 2). The examiners (MSS and PF), one (PF) more experienced in diagnosis/treatment of TMDs patients than the other, were tested in comparison to a “reference examiner” (gold standard). This person was an instructor and teacher at the Section of Clinical Oral Physiology, trained in the consortium guidelines, and also the contact person for the DC/TMD course. In 2013, the early edition of the protocol “Diagnostic Criteria for TMDs, Clinical Protocol and Assessment Instruments” [13] was sent in the English version to the examiners two weeks preceding the examination session. The purpose was that the examiners would be able to learn and memorize the verbal commands previous to an eight-hour training and calibration course. Then, the protocol was implemented in a total of 16 participants including a 1 : 4 ratio of healthy/symptomatic individuals. The healthy participants originated from the patient catchment area of Aarhus University while those with a mix of muscular and joint problems were recruited among the TMD patients at the Section's clinic. In 2014, the same examiners before the examination session was conducted were encouraged to recapture the instructions and verbal commands that had been taught the year before and clarify any information related to the examination protocol. This could be done by e-mail contact or at a prior 45-minute session at the day of the clinical examination. Also, this year there were 16 participants. In both years, the same assessment procedures and parameters were used [13]. It was ensured that recorders assisted the examiners to complete the DC/TMDs examination form and that the examiners were blind to the participant's previous examinations or medical-dental history.

### 2.1. The Examination Procedure

The time requirement at both sessions was set to 20 minutes per examination. Four examination rounds were organised during a day, each round with four participants, which also allowed for regular breaks between the time sections. An in advance “Order of Examination Sheet” was conducted, both to assure examiner rotation in order to avoid examiner sequence could influence the results and to ensure that each participant was examined by each examiner. If this had not been taken into account, bias could have occurred as participants at the end of the series of examinations might have presented a more tensed or more stressed musculature. During the examinations, the participants who were offered a fee for participating sat comfortably upright in chairs that could be adjusted for height. The examiners stood to the right of the participants, facing them, but position changes were allowed if needed.

### 2.2. Measurements

The sequence of the examination process was as follows: firstly, information about pain and headache location during the last 30 days was requested, recorded as 0 (No: no pain) and 1 (Yes: pain). The subsequent registered measurements of the mandible were opening pattern, opening movements (pain-free opening, maximum-unassisted opening, and maximum-assisted opening), lateral (right lateral and left lateral) and protrusive movements, TMJ noises during opening, closing, lateral and protrusive movements, and joint locking. Pain during palpation of the TMJ and on supplemental muscles was the last measure. For accurate muscle palpation prior to the palpation examinations, finger pressure was calibrated by an appropriate force-measuring device (Palpeter®, Dentrade, Köln, Germany); 1 kg finger pressure for the masseter muscle (three horizontal zones: origin, body, and insertion of the masseter) and temporalis muscle (three vertical zones: anterior, middle, and posterior as well as around the lateral joint pole); 0.5 kg finger pressure for the lateral joint pole and for supplemental muscles. The palpation pressure was held for two seconds to determine pain and for five seconds to record a referred pain, two seconds for muscle palpation and finally, five seconds for lateral joint pole and around lateral joint pole.

### 2.3. Statistical Methods

A set of reliability coefficients for the clinical measurements were used. Interexaminer agreements between the “reference” and each of the two examiners (MSS and PF) of the clinical continuous data were assessed by applying Bland–Altman plots with limits of agreement (LoA) and intraclass correlation coefficients (ICC). For clinical categorical data with “Yes” and “No” responses, pain based on muscle palpation and joint sounds, kappa statistic (unweighted Cohen's kappa), and percent agreement with the “reference” were calculated. Comparison of percent agreement with the “reference” between Examiner 1 and Examiner 2 was done separately for 2013 and 2014, using McNemar's test in order to take into account that both examiners evaluated the same set of patients. Comparison of percent agreement between 2013 and 2014 was done using chi-squared tests since the patient samples in 2013 and 2014 were independent. The level of statistical significance was set to 5 percent. All analyses were undertaken with SPSS 24 (IBM Corp., Armonk, NY), and the graphics were derived using Matlab 9.0 (The MathWorks Inc., Natick, MA) to evaluate the interexaminer agreement.

## 3. Results

### 3.1. Measurement of Mandibular Range of Motion

**Figure 1:**
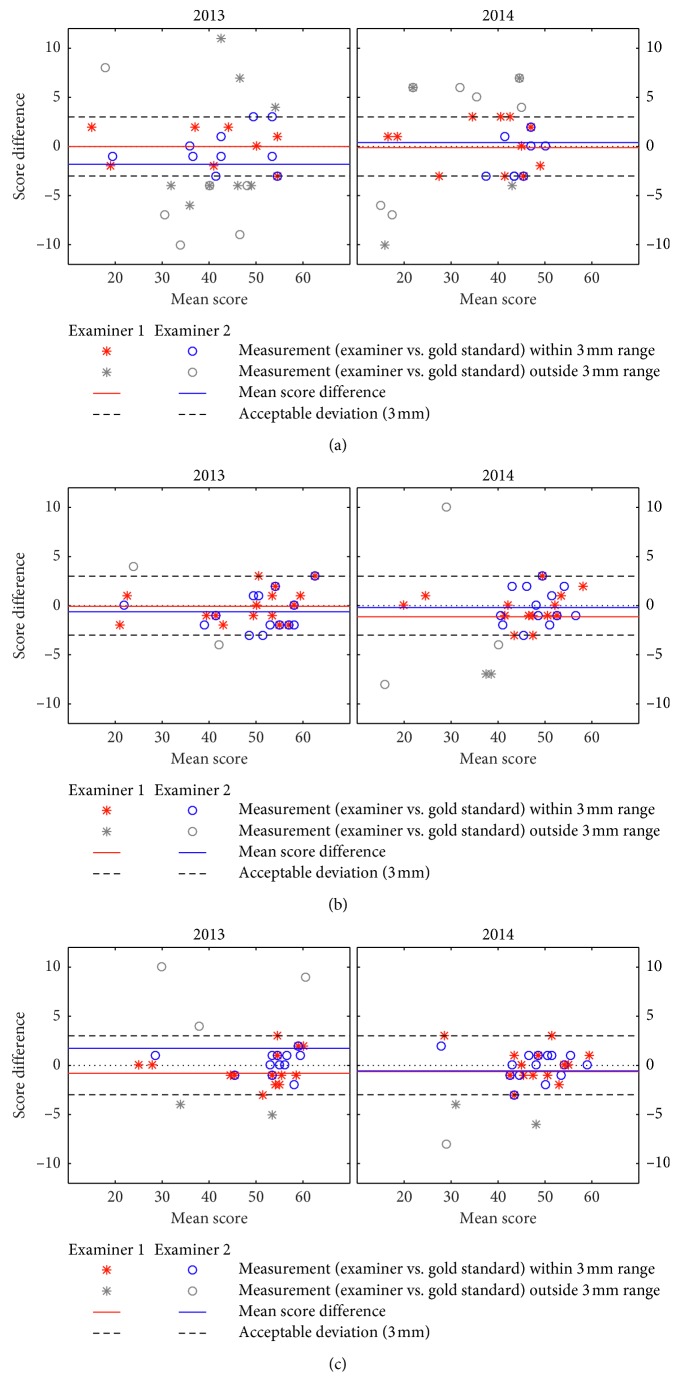
Opening movements ((a) pain-free opening, (b) maximum-unassisted opening, and (c) maximum-assisted opening). Bland–Altman plot for two examiners (raters) versus the “reference” (gold standard) at each time points (2013 and 2014). Mean scores in mm.

**Figure 2:**
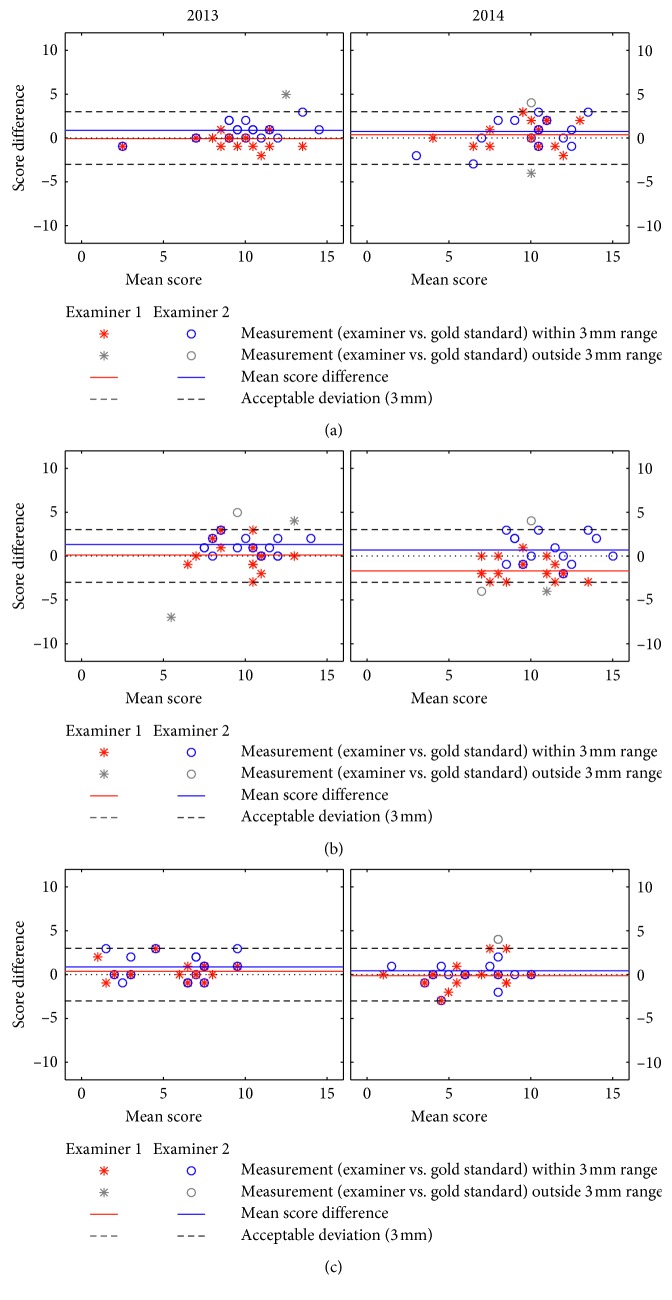
Lateral and protrusive movements ((a) right lateral, (b) left lateral, and (c) protrusion) at both sites for Bland–Altman plot for two examiners (raters) versus the “reference” (gold standard) at each time point (2013 and 2014). Mean scores in mm.

Comparisons of the two trainee examiners (MSS and PF) in measuring the mandibular range of motion with the “reference” in 2013 and in 2014 are presented in Figure 1 (pain-free opening, maximum-unassisted opening, and maximum-assisted opening). Comparisons in respect of lateral and protrusive movements are presented in Figure 2 (right lateral, left lateral, and protrusion). Observation agreement of examiners vs. “reference” within a three-millimeter range was more frequent in the opening movements of maximum-unassisted opening and of maximum-assisted opening than in the opening movement of pain-free opening. The acceptable three-millimeter deviation was revealed by both examiners for maximum-unassisted opening, maximum-assisted opening, right lateral, left lateral and, protrusion both in 2013 and 2014.  Table 1 presents interexaminer reliabilities using ICC values (average measures) and shows that the level of ICC for all measurements based on comparison between each examiner with “reference,” was above 0.75 except for left lateral measurement (ICC: 0.60). For opening movements, the ICC values in 2013 and 2014 were almost identical for both trainee examiners. As for lateral at both sites and protrusive movements, the reliability scores varied depending on whether they were lower, at the same level, or higher in 2014 than in 2013. No clear and consistent change of the agreement from 2013 to 2014 could therefore be registered. Between the examiners, sometimes Examiner 1 had the higher ICC values; other times it was Examiner 2. Due to this variation between the examiners in reporting the highest reliability scores when compared to “reference,” no clear difference in agreement between them could be observed.

### 3.2. Measurements Based on Muscle Palpation

Table 2 is descriptive and presents by Cohen's kappa scores and consistency values of the achieved interexaminer agreement between the “reference” and each of the two examiners for registering pain upon muscle palpation (Yes/No). The majority of the present percent agreement scores achieved did not significantly change from 2013 to 2014 (Supplemental Table 1). However, the percent agreement achieved when each examiner was compared to the “reference” showed statistical difference from 2013 to 2014 in two muscle zones (Examiner 1: body of m. masseter and Examiner 2: posterior zone of m. temporalis). Supplemental Table 1 shows that Examiner 1 experienced higher value (*p* value: 0.049), and Examiner 2 experienced lower value (*p* value: 0.042). Between the examiners, only in 2014, for palpation of m. masseter origin zone, statistical difference (*p* value: 0.022) in percent agreement could be shown (Examiner 1 had the highest reliability value). Additionally for Examiner 1, percent agreement for TMJ sounds in the form of clicking during closing movement significantly improved (*p* value: 0.039) from 2013 to 2014 (Supplemental Table 1).

Crepitus was infrequently observed. The “reference” did not register crepitus during the Opening movement in 2013, consistent with the other examiners (100% agreement). In 2014, however, the “reference” registered one crepitus during the Closing movement, not registered by the trainee examiners. Two cases of crepitus during lateral and protrusive movements registered by the “reference” were not noticed by the trainee examiners. On the contrary, both trainee examiners recorded crepitus in the same participant, but this was not observed by the “reference.”

## 4. Discussion

This is the first study, as far as we know, that analyses the reliability of repeat measuring of components in the recently introduced DC/TMD diagnostic tool. Publications in the literature so far have been about whether DC/TMDs can be considered a valid screener for detecting TMDs, and whether it is a valid diagnostic criterion for different TMD subgroups [10, 24]. Due to the fact that reliability studies focusing on this new DC/TMD tool are so far few, this type of research should be appreciated.

The present study assesses the interexaminer reliability between the “reference” and each of the examiners in the DC/TMDs, and Axis I examination method failed to demonstrate any consistently clear change after recalibration; e.g., improvement as reported when the RDC/TMD was chosen as a diagnostic tool [17]. Only sporadical statistical differences were registered. Therefore, for the majority of the clinical measures, the null hypothesis could not be rejected.

The level of ICC for all but one mandibular movement was above 0.75, which was considered excellent [26]. The one registration below 0.75 of the left-lateral movement in 2013 (ICC: 0.60) was categorised as good. Interestingly, both examiners had their lowest ICC values when assessing the left-lateral movement. One explanation might be that, while standing to the right in front of the participant, it may be easier to register the movement to the right than to the left. The highest ICC values reported when the examiners were compared with the “reference” were for maximum-assisted or maximum-unassisted opening movements; this in line with other authors [18, 27].

A common method for detecting muscle tenderness is manual palpation [28]. Low agreement among examiners when examining the origin of the masseter has been reported as being a particular problem [28], a zone which also in this study showed some poor reliability values. Using standardization of palpation pressure, in spite of what was expected, did not contribute to a high level of reliability. One explanation for the relatively low reliability values for some zones of muscles might be that as many as 75% of recruited patients were TMD patients. Perhaps, applying the Palpeter standardization instrument directly on the muscle sites would have given higher reliability results.

The examiner agreement concerning the detection of click noises was consistent with a previous study of John and Zwijnenburg [18]. Examiner 1 could also show significant improvement in clicking during closing movement from 2013 to 2014. In spite of the high TMD prevalence among participants, crepitation was infrequent. The “reference” only found it once in 2014, and the examiners did not catch it. Therefore, the present extremely high percentage of agreement among the examiners with respect to crepitus most probably would have been lower if more participants had displayed it. The use of Cohen's kappa in measuring crepitus could not be used because of difficulties in interpreting the result. The underlying cause was the combination of extremely low prevalence of one of the decisions and a low sample size.

Leher et al. [20] have argued that examiner calibration rather than professional experience is the most important factor for reliable measurements of TMD symptoms. In this study, it seemed that prior experience was of lesser importance. However, the importance of clinical experience in deciding appropriate TMD diagnosis and how to treat it (outside the scope of this article) should be mentioned. Despite the ability to register clinical findings correctly, these must be combined with the appropriate imaging and other diagnostic tools to allow a correct diagnosis and treatment plan. Salloch and coworkers [29] have recently stressed the importance of the physician's expertise to find appropriate diagnoses and treatment plans for each patient in oncologic decision-making. Registration of pathology in the TMJ, including jaw movements, muscle palpation, and TMJ noises, may be seen either independently or in combination with TMDs such as myalgia, disc derangement, or inflammatory joint diseases. Therefore, it is essential that the examiner has clinical experience to make the appropriate diagnosis and treatment plan for each patient. Similar reliability between the examiners in measuring jaw movements, muscle palpation, and TMJ sounds does not always imply proper diagnosis and treatment of different TMDs but a more complex issue requiring clinical experience and knowledge of the examiner.

Self-instruction for examiners in DC/TMDs, according to Vilanova et al. [25], has been reported to be as effective as an examiner course. Explanations for this finding may be that the instructions for DC/TMD examinations are clear and easily memorised. This could also explain why the majority of the reliability coefficients in the present study did not change after recalibration such as when RCD/TMDs were applied [17].

### 4.1. Limitations

The instructions used in the DC/TMD protocol for the participants were provided in English, a possible source of misunderstanding as the first language of participants was Danish. However, none of the participants showed any sign of failure to understand the instructions. The reason for using the English language was that a back-translated version was not yet available. Another limitation was the relatively small sample size resulting in low power for statistical tests and that the examiners sometimes had problems recording all (*n*=32) muscle palpation sites, especially in 2013. An explanation for better managing of the time schedule in 2014 could be that the examiners were more experienced.

## 5. Conclusion

No clear and consistent difference in the level of agreement between the two examiners could be observed, although one was more experienced than the other. Likewise, for most components of the DC/TMD tool, recalibration of examiners did not change the reliability findings.

The present findings underline that DC/TMDs are simple and well defined, having operational definitions with clear presentations. However, these findings should be further investigated in longitudinal clinical cohort studies using the DC/TMD protocol.

## Figures and Tables

**Table 1:** Reliability (intraclass correlation coefficients (ICC)) for opening, lateral, and protrusive movements (mm). ICC calculations based on ICC values between the “reference” and Examiner 1 and the “reference” and Examiner 2 (average measurements). For ICC interpretation, https://www.ncbi.nlm.nih.gov/pmc/articles/PMC4913118/.

	2013		2014	
	Examiner 1	Examiner 2	Examiner 1	Examiner 2
Opening movements				
Pain-free opening	0.96 (0.89, 0.99)	0.96 (0.88, 0.99)	0.97 (0.91, 0.99)	0.96 (0.88, 0.99)
Maximum-unassisted opening	0.99 (0.99, 0.99)	0.99 (0.98, 0.99)	0.98 (0.95, 0.99)	0.96 (0.90, 0.99)
Maximum-assisted opening	0.99 (0.97, 0.99)	0.97 (0.92, 0.99)	0.98 (0.94, 0.99)	0.98 (0.94, 0.99)
Lateral at both sites and protrusive movements				
Right lateral	0.91 (0.73, 0.97)	0.96 (0.90, 0.99)	0.81 (0.46, 0.93)	0.87 (0.63, 0.95)
Left lateral	0.60 (-0.16, 0.86)	0.88 (0.65, 0.96)	0.80 (0.68, 0.96)	0.77 (0.33, 0.92)
Protrusion	0.96 (0.88, 0.99)	0.92 (0.76, 0.97)	0.90 (0.70, 0.96)	0.85 (0.56, 0.95)

**Table 2:** Cohen's kappa (K) and examiner agreement (% agreement values in parentheses) for reporting pain (Yes/No) and for reporting TMJ noises during opening and closing movements (Yes/No) between the “reference” and Examiner 1 and the “reference” and Examiner 2. *N* = number of observations.

	2013				2014			
	Examiner 1		Examiner 2		Examiner 1		Examiner 2	
	*N*	*K* (% agreement)	*N*	*K* (% agreement)	*N*	*K* (% agreement)	*N*	*K* (% agreement)
M. temporalis^1^								
Posterior	24	0.78 (91.7)	27	0.81 (92.6)	32	0.40 (78.1)	32	0.30 (71.9)
Middle	23	0.56 (78.3)	26	0.33 (65.4)	31	0.48 (74.2)	32	0.57 (81.3)
Anterior	21	0.35 (66.7)	27	0.70 (85.2)	32	0.68 (84.4)	32	0.56 (78.1)
M. masseter^2^								
Origin	24	0.50 (75.0)	26	0.34 (65.4)	31	0.69 (80.6)	31	0.27 (64.5)
Body	22	0.31 (68.3)	25	0.66 (88.0)	30	0.74 (90.0)	32	0.67 (84.3)
Insertion	20	0.50 (75.0)	26	0.57 (76.9)	30	0.67 (83.3)	32	0.87 (93.8)
TMJ sounds								
Open/close^3^								
Click								
Open	32	0.48 (75.0)	32	0.54 (78.1)	32	0.62 (90.6)	32	0.72 (93.8)
Close	32	0.69 (87.5)	32	0.72 (87.5)	32	1.00 (100.0)	32	0.72 (96.9)
Crepitus								
Open	32	(100.0)	32	(100.0)	32	(100.0)	32	(100.0)
Close	32	(100.0)	32	(93.8)	32	(96.9)	32	(96.9)
Lateral/protrusive^4^								
Click	32	0.66 (84.4)	32	0.62 (81.3)	32	0.53 (87.5%)	32	0.53 (87.5)
Crepitus	32	(100.0)	32	(100.0)	32	(90.6)	32	(90.6)

^1^Three vertical zones together (both sides). ^2^Three horizontal zones together (both sides). ^3^Opening and closing movements (both sides). ^4^Lateral and protrusive movements (both sides). For interpretation of the kappa statistic, https://www.ncbi.nlm.nih.gov/pmc/articles/PMC3900052/.

## Data Availability

The data used to support the findings of this study are available from the corresponding author upon request. The data are collected in paper format and in Excel.

## Supplementary Materials

(Supplementary Materials) Supplemental Table 1: it presents both *p* values based on comparison of percent agreement between Examiner 1 and Examiner 2 separately for 2013 and 2014 and p-values based on comparison of percent agreement between 2013 and 2014.
