# Supplementary material for: DC/TMD Examiner Protocol: Longitudinal Evaluation on Interexaminer Reliability
Source: Pain Res Manag. 2018 Sep 26;2018:7474608. doi: 10.1155/2018/7474608 (PMC6178177; doi:10.1155/2018/7474608)
Supplement: (Supplementary Materials) — Supplemental Table 1: it presents both p values based on comparison of percent agreement between Examiner 1 and Examiner 2 separately for 2013 and 2014 and p-values based on comparison of percent agreement between 2013 and 2014. [file 7474608.f1.docx]

Supplemental Table 1 presents p-values from comparison measuring between percent agreement between Examiner 1 and Examiner 2 separately for 2013 and 2014 (Part 1) The other comparison measurement is percent agreement between 2013 and 2014 (Part 2).

|  |  |  |  | 2013 | | | | |  | 2014 | | | | | | |  |
| --- | --- | --- | --- | --- | --- | --- | --- | --- | --- | --- | --- | --- | --- | --- | --- | --- | --- |
|  |  |  |  | Examiner 1 | |  | Examiner 2 | |  | Examiner 1 | |  | Examiner 2 | | |  |  |
|  |  |  |  | Total | N (%) |  | Total | N (%) p-value | | Total | N (%) |  | Total | | N (%) | p-value |  |
| M. temporalis^1^ | | | |  |  |  |  |  |  |  |  |  |  |  | | |  |
|  | Posterior | |  | 24 | 22 (91.7%) |  | 27 | 25 (92.6%) 1.000 | | 32 | 25 (78.1%) |  | 32 |  | 23 (71.9%) 0.727 | |  |
|  | Middle | |  | 23 | 18 (78.3%) |  | 26 | 17 (65.4%) 0.219 | | 31 | 23 (74.2%) |  | 32 |  | 26 (81.3%) 0.508 | |  |
|  | Anterior | |  | 21 | 14 (66.7%) |  | 27 | 23 (85.2%) 0.687 | | 32 | 27 (84.4%) |  | 32 |  | 25 (78.1%) 0.687 | |  |
| M. masseter^2^ | | |  |  |  |  |  |  |  |  |  |  |  |  |  |  |  |
|  | Origin | |  | 24 | 18 (75.0%) |  | 26 | 17 (65.4%) 0.250 | | 31 | 25 (80.6%) |  | 31 |  | 20 (64.5%) 0.022 * | |  |
|  | Body | |  | 22 | 15 (68.3%) |  | 25 | 22 (88.0%) 0.250 | | 30 | 27 (90.0%) |  | 32 |  | 27 (84.3%) 1.000 | |  |
|  | Insertion | |  | 20 | 15 (75.0%) |  | 26 | 20 (76.9%) 1.000 | | 30 | 25 (83.3%) |  | 32 |  | 30 (93.8%) 0.375 | |  |
| TMJ sounds | | |  |  |  |  |  |  |  |  |  |  |  |  |  |  |  |
|  | Open/close^3^ | | |  |  |  |  |  |  |  |  |  |  |  |  |  |  |
|  |  | Click | |  |  |  |  |  |  |  |  |  |  |  |  |  |  |
|  |  |  | Open | 32 | 24 (75.0%) |  | 32 | 25 (78.1%) 1.000 | | 32 | 29 (90.6%) |  | 32 |  | 30 (93.8%) 1.000 | |  |
|  |  |  | Close | 32 | 28 (87.5%) |  | 32 | 28 (87.5%) 1.000 | | 32 | 32 (100.0%) |  | 32 |  | 31 (96.9%) - | |  |
|  |  | Crepitus | |  |  |  |  |  |  |  |  |  |  |  |  |  |  |
|  |  |  | Open | 32 | 32 (100.0%) |  | 32 | 32 (100.0%) 1.000 | | 32 | 32 (100.0%) |  | 32 |  | 32 (100.0%) 1.000 | |  |
|  |  |  | Close | 32 | 32 (100.0%) |  | 32 | 30 (96.9%) - | | 32 | 31 (96.9%) |  | 32 |  | 31 (96.9%) 1.000 | |  |
|  | Lateral/protrusive^4^ | | |  |  |  |  |  |  |  |  |  |  |  |  |  |  |
|  |  | Click | | 32 | 27 (84.4%) |  | 32 | 26 (81.3%) 1.000 | | 32 | 28 (87.5%) |  | 32 |  | 28 (87.5%) 1.000 | |  |
|  |  | Crepitus | | 32 | 32 (100.0%) |  | 32 | 32 (100.0%) 1.000 | | 32 | 29 (90.6%) |  | 32 |  | 29 (90.6%) 1.000 | |  |
| ^1^3 vertical zones together (both sides). ^2^3 horizontal zones together (both sides). ^3^Opening and closing movements (both sides). ^4^Lateral and protrusive movements (both sides). *: p-value below 0.05. | | | | | | | | | | | | | | | | |  |
|  |  |  |  |  |  |  |  |  |  |  |  |  |  |  |  |  |  |
|  |  |  |  |  |  |  |  |  |  |  |  |  |  |  |  |  |  |
|  |  |  |  |  |  |  |  |  |  |  |  |  |  |  |  |  |  |
|  |  |  |  | Examiner 1 | | | | |  | Examiner 2 | | | | | | |  |
|  |  |  |  | 2013 | |  | 2014 | |  | 2013 | |  | 2014 | | | |  |
|  |  |  |  | Total | N (%) |  | Total | N (%) p-value | | Total | N (%) |  | Total | | N (%) | p-value |  |
| M. temporalis^1^ | | | |  |  |  |  |  |  |  |  |  |  |  |  |  |  |
|  | Posterior | |  | 24 | 22 (91.7%) |  | 32 | 25 (78.1%) | 0.172 | 27 | 25 (92.6%) |  | 32 |  | 23 (71.9%) | 0.042 * | |
|  | Middle | |  | 23 | 18 (78.3%) |  | 31 | 23 (74.2%) | 0.730 | 26 | 17 (65.4%) |  | 32 |  | 26 (81.3%) | 0.170 |  |
|  | Anterior | |  | 21 | 14 (66.7%) |  | 32 | 23 (84.4%) | 0.686 | 27 | 23 (85.2%) |  | 32 |  | 25 (78.1%) | 0.488 |  |
| M. masseter^2^ | | |  |  |  |  |  |  |  |  |  |  |  |  |  |  |  |
|  | Origin | |  | 24 | 18 (75.0%) |  | 31 | 25 (80.6%) | 0.615 | 26 | 17 (65.4%) |  | 31 |  | 20 (64.5%) | 0.945 |  |
|  | Body | |  | 22 | 15 (68.3%) |  | 30 | 27 (90.0%) | 0.049 * | 25 | 22 (88.0%) |  | 32 |  | 27 (84.3%) | 0.696 |  |
|  | Insertion | |  | 20 | 15 (75.0%) |  | 30 | 25 (83.3%) | 0.470 | 26 | 20 (76.9%) |  | 32 |  | 30 (93.8%) | 0.065 |  |
| TMJ sounds | | |  |  |  |  |  |  |  |  |  |  |  |  |  |  |  |
|  | Open/close^3^ | | |  |  |  |  |  |  |  |  |  |  |  |  |  |  |
|  |  | Click | |  |  |  |  |  |  |  |  |  |  |  |  |  |  |
|  |  |  | Open | 32 | 24 (75.0%) |  | 32 | 29 (90.6%) | 0.098 | 32 | 25 (78.1%) |  | 32 |  | 30 (93.8%) | 0.072 |  |
|  |  |  | Close | 32 | 28 (87.5%) |  | 32 | 32 (100.0%) | 0.039 * | 32 | 28 (87.5%) |  | 32 |  | 31 (96.9%) | 0.162 |  |
|  |  | Crepitus | |  |  |  |  |  |  |  |  |  |  |  |  |  |  |
|  |  |  | Open | 32 | 32 (100.0%) |  | 32 | 32 (100.0%) | | 32 | 32 (100.0%) |  | 32 |  | 32 (100.0%) | |  |
|  |  |  | Close | 32 | 32 (100.0%) |  | 32 | 31 (96.9%) | 0.313 | 32 | 30 (93.8%) |  | 32 |  | 31 (96.9%) | 0.554 |  |
|  | Lateral/protrusive^4^ | | |  |  |  |  |  |  |  |  |  |  |  |  |  |  |
|  |  | Click | | 32 | 27 (84.4%) |  | 32 | 28 (87.5%) | 0.719 | 32 | 26 (81.3%) |  | 32 |  | 28 (87.5%) | 0.491 |  |
|  |  | Crepitus | | 32 | 32 (100.0%) |  | 32 | 29 (90.6%) | 0.076 | 32 | 32 (100.0%) |  | 32 |  | 29 (90.6%) | 0.090 |  |
| ^1^3 vertical zones together (both sides). ^2^3 horizontal zones together (both sides). ^3^Opening and closing movements (both sides). ^4^Lateral and protrusive movements (both sides). *: p-value below 0.05. | | | | | | | | | | | | | | | | |  |
